# Comparative study of sculptured metallic thin films deposited by oblique angle deposition at different temperatures

**DOI:** 10.3762/bjnano.9.89

**Published:** 2018-03-22

**Authors:** Susann Liedtke, Christoph Grüner, Jürgen W Gerlach, Bernd Rauschenbach

**Affiliations:** 1 Leibniz Institute of Surface Engineering (IOM), Permoserstraße 15, D-04318 Leipzig, Germany

**Keywords:** biaxial texture, metallic tilted columns, oblique angle deposition, porosity, shadowing, thin films

## Abstract

Metals with a wide range of melting points are deposited by electron beam evaporation under oblique deposition geometry on thermally oxidized Si substrates. During deposition the sample holder is cooled down to 77 K. It is observed that all obliquely deposited metals grow as tilted, high aspect ratio columns and hence with a similar morphology. A comparison of such columns with those deposited at room temperature (300 K) reveals that shadowing dominates the growth process for columns deposited at 77 K, while the impact of surface diffusion is significantly increased at elevated substrate temperatures. Furthermore, it is discussed how the incidence angle of the incoming particle flux and the substrate temperature affect the columnar tilt angles and the porosity of the sculptured thin films. Exemplarily for tilted Al columns deposited at 77 K and at 300 K, in-plane pole figure measurements are carried out. A tendency to form a biaxial texture as well as a change in the crystalline structure depending on the substrate temperature is found for those films.

## Introduction

The ability to produce highly porous metallic thin films is a substantial issue for a large number of applications [1]. For instance, such thin films are the basis for surface enhanced Raman sensors, which are highly sensitive in the detection of environmental toxics [2] or glycated hemoglobin [3]. It has also been shown that highly porous metallic thin films can be used to improve the electrode’s performance for applications in fuel cells [4–6] or Li-ion batteries [7–8]. Oblique angle deposition (OAD) [9–11] opens the opportunity to grow such films in an elegant and easy to handle way. During the OAD process, the substrate is tilted to an oblique incidence angle θ between incoming particle flux and normal of the substrate so that shadowing is induced during the growth process. This leads to the development of three dimensionally separated tilted metallic columns that grow over a large substrate area. During deposition, single metal atoms condense on the substrate surface and form microscopic nuclei. Due to the oblique deposition geometry, those nuclei create a shadowed region, where the other incoming particles cannot condense. Ballistic shadowing forces the growing nuclei to develop in separated, tilted columns oriented towards the particle source. However, metal atoms show already at room temperature an enlarged surface mobility, which enables them to diffuse also in the shadowed regions. Consequently, the shadowing effect is smeared out, which reduces the control of the shaping of the tilted columns and leads to the growth of more compact thin films. To overcome this, the influence of surface diffusion has to be limited. So far, metallic (Ag and Au) tilted columns grown at low substrate temperatures (133 K) have exclusively been studied by Jen et al. [12–14].

In the present study, the growth processes of seven different metals (Al, Ni, Ti, Co, Cr, Mo and Ta) deposited at 77 K are compared with each other and, based on this comparison, more general conclusions are drawn for the growth of metallic thin films in general. This is of interest, because a consolidated knowledge of the growth processes is a basic requirement to finally design, tune and optimize the properties of the metallic thin films. Besides columnar morphology, the relation between tilt angle β, porosity P and incidence angle θ is in focus of this paper. Further, the influence of surface diffusion and changes in the crystalline structure are studied.

## Results and Discussion

### Morphology and texture

**Figure 1:**
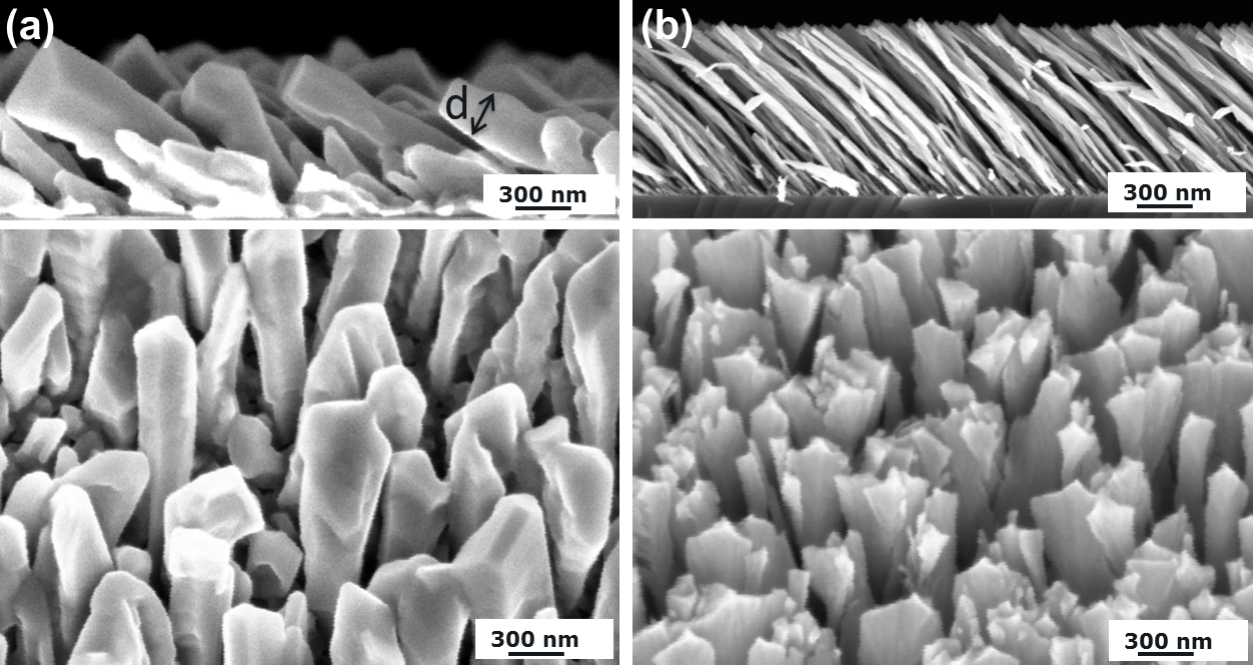
Cross-sectional (top) and top-view (bottom) SEM images of tilted Al columns deposited at an incidence angle of 82° on thermally oxidized Si substrates and at a substrate temperature of (a) 300 K and (b) 77 K.

Figure 1 shows tilted Al columns that are deposited obliquely at an incidence angle of 82° and a substrate temperature of 300 K (left) and at 77 K (right). Especially the cross-sectional SEM images in Figure 1 reveal significant morphological differences. Al columns deposited at 300 K exhibit a columnar diameter *d* = 126 ± 25 nm, approximately. For those columns grown at 77 K, the columnar diameter is about 36 ± 10 nm, which is a reduction by a factor of approximately three. To understand these remarkable morphological changes, the influence of surface diffusion on the growth process of metallic columns has to be taken into account. For Al, it is known that this metal has high surface adatom mobility already at room temperature [15]. For this reason, the incoming Al atoms are sufficiently mobile to move also in the shadowed regions of the columns. Consequently, the shadowing effect is reduced and the entire column becomes broader in diameter. However, as the substrate is cooled down to 77 K, the mobility of the incoming Al atoms on the column surface is significantly reduced. This supports the growth of columns with a high aspect ratio. To conclude, the substrate temperature and in turn surface diffusion have a significant influence on the growth of tilted Al columns.

Recently, it was demonstrated that metal columns (such as Ti and Cr) grown by oblique angle deposition have a crystalline structure at 300 K [16]. In the present study, this result can be confirmed for the metal Al grown by OAD at 77 K and 300 K. In-plane pole figure measurements are carried out exemplarily for Al thin films to study the orientation of these crystallites.

**Figure 2:**
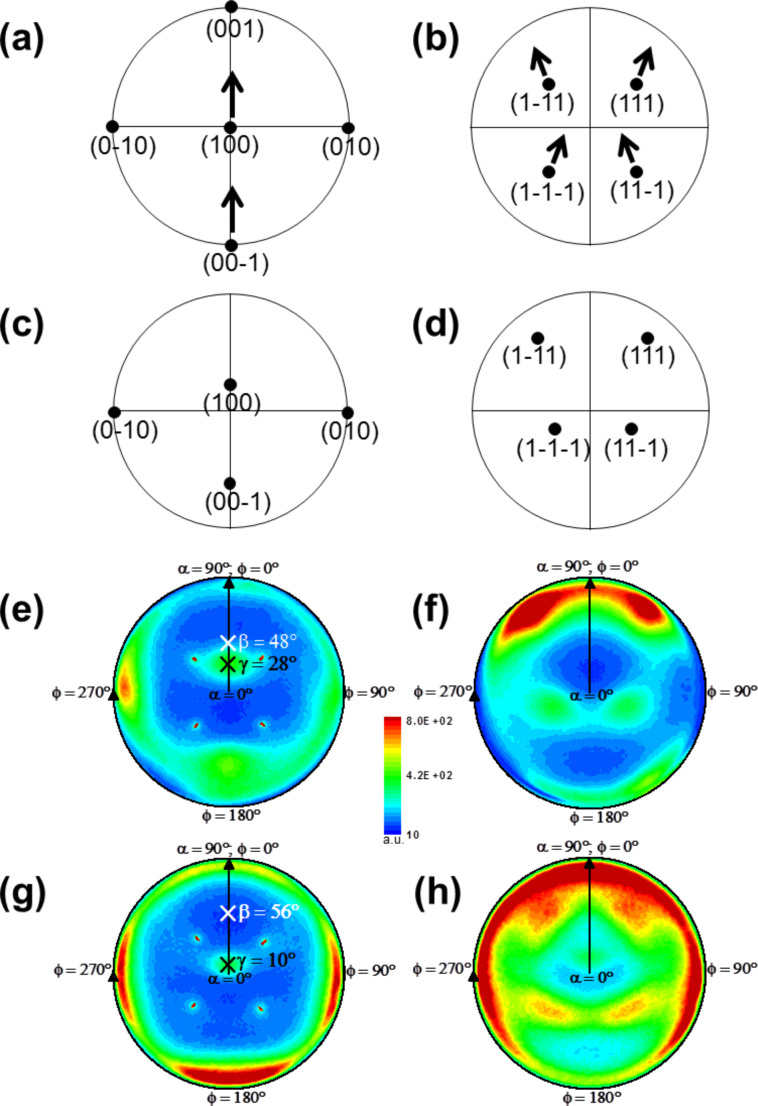
(a) Calculated in-plane pole figures for the cubic Al{200} and (b) Al{111} planes in a vertically (θ = 0°) deposited thin film. The black arrows illustrate that the pole density maxima move as the substrate is tilted to oblique angles (compare (c)–(h)). (c) Calculated in-plane pole figures for the cubic Al{200} and (d) Al{111} planes in an obliquely (θ = 82°) deposited thin film. Measured in-plane pole figures for the cubic (e) Al{200} and (f) Al{111} planes in an obliquely (θ = 82°) deposited thin film at 77 K. (g) Al{200} and (h) Al{111} planes in an obliquely (θ = 82°) deposited thin film at 300 K. The direction of the crystallites is γ and the tilt angle of the columns is β, both measured with respect to the substrate normal (see (e) and (g)). Intensity (a.u.) is depicted on a linear scale (see scale bar) and applies for all measured in-plane pole figures ((e)–(h)).

Stereographic projections of the in-plane pole figures for such films are calculated [17]. Thereby, Figure 2a and 2b depict the calculated stereographic projections for a vertically deposited (θ = 0°) thin Al film with the preferred growth direction [100] (compare center pole density maximum in Figure 2a). The (1−11), (111), (1−1−1) and (11−1) pole density maxima in Figure 2b represent the four space diagonals in the cubic Al unit cell. The black arrows illustrate that when the sample is tilted to an oblique angle θ = 82° the pole density maxima in Figure 2a and 2b move (see calculations in Figure 2c and 2d).

Experimentally, in-plane pole figure measurements of Al columns deposited on thermally oxidized Si substrates at θ = 82° are carried out using the Al(111) and Al(200) Bragg reflections at 2θ = 38.472° and 2θ = 44.738°, respectively. Figure 2e and 2f as well as Figure 2g and 2h show the stereographic projections of these measurements for substrate temperatures of 77 K and 300 K, respectively. The center (200) pole density maxima in Figure 2e and 2g indicate that all [100] directions of the Al crystallites are oriented in the same direction. Additionally, in Figure 2f and 2h four broad but still separated pole density maxima at a polar angle α = 54.7° are obtained. Notice that the underlying symmetry of Al is cubic. Consequently, this experimentally observed angle is in good agreement to the expected angle between (100) and (111) lattice planes. The pole density maxima are separated by an azimuthal angle φ = 90°. This well-defined pole density distribution reveals a mostly biaxial texture of the Al columns. There is also a low density ring connecting each two pole density maxima, revealing a polycrystalline fiber-texture contribution. The four sharp peaks in Figure 2e and 2g, respectively, originate from the Si(100) substrate. The presence of an 800 nm thick oxide layer on the substrate prohibits an epitaxial relationship between the columns and the substrate. In summary, it can be noted that the calculated positions of the pole density maxima are in good agreement with the measured positions of the pole density maxima.

**Figure 3:**
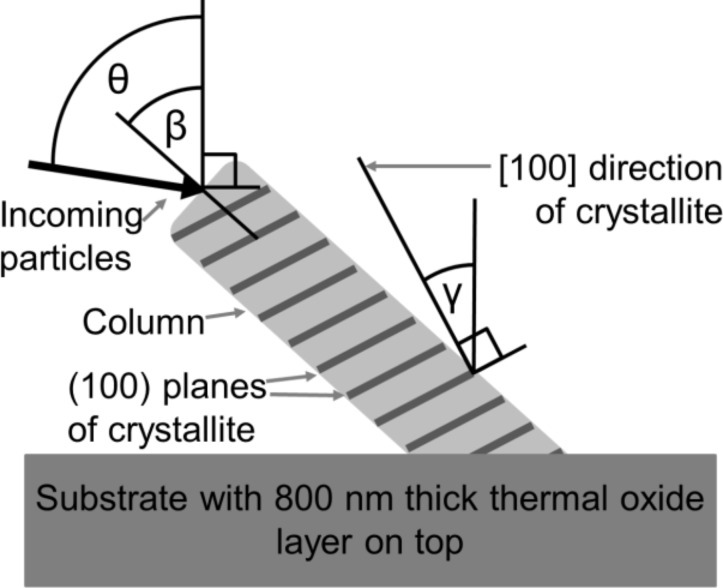
Schematic illustration of a tilted Al column deposited on a thermally oxidized Si substrate at an oblique angle θ = 82°. The column is tilted by the angle β. The [100] directions of the crystallites are represented by γ. All angles are measured with respect to the substrate normal.

Furthermore, Figure 2e and 2g reveal that the [100] direction of the crystallites γ differs from the tilt angle β of the columns, both measured with respect to the substrate normal. For the columns deposited at 77 K, the tilt angle is β = 48 ± 3°, whereas the [100] direction of the crystallites is γ = 28° tilted away from the substrate normal. Consequently, there is an angular difference of approximately 20° (48° − 28°) between tilt angle of the column and [100] direction of the crystallites. The columns deposited at 300 K have a tilt angle β = 56 ± 3°, while the [100] direction of the crystallites is inclined by γ = 10°. Thus, the angular difference obtained is 46° (56° − 10°). For illustration see Figure 3.

Besides these obliquely deposited Al thin films (θ = 82°), polycrystalline Al thin films are grown with a vertically incoming Al atom flux (θ = 0°). The θ–2θ scans (not shown here) of these vertically deposited films reveal a preferred [111] growth direction for deposition at 77 K, but [111] as well as [100] growth directions for deposition at 300 K. According to Wulff´s rule [18], the lowest surface energy plane of fcc Al is the (111) plane. Typically, this is the plane with the highest surface diffusion, which finally develops to the faceted surface of the crystallite under equilibrium conditions.

In summary, differences in the crystalline texture of the Al columns are observed for oblique deposition at 77 K and 300 K. Besides, there are differences in the preferred growth directions found for vertically and obliquely deposited thin Al films. These findings indicate that substrate temperature and in turn surface diffusion as well as the direction of the incoming particle flux affect the crystalline texture of the grown films.

**Figure 4:**
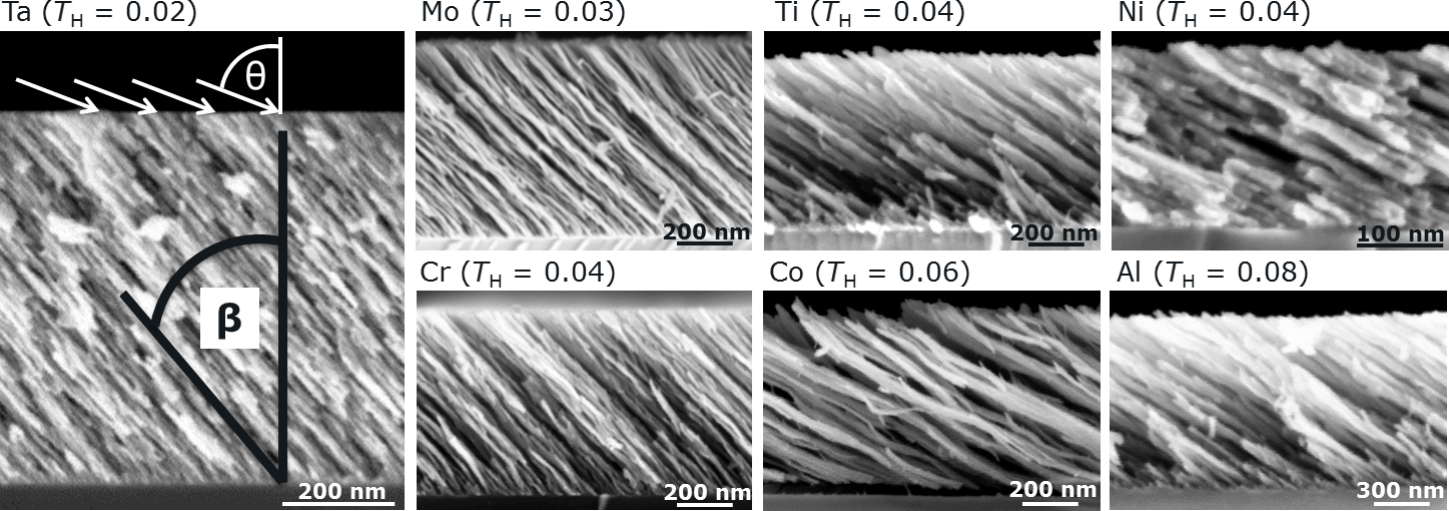
Cross-sectional SEM images of tilted metallic columns deposited at an incidence angle of 82° on thermally oxidized Si substrates and at a substrate temperature of 77 K. The tilt angle of the columns is β and θ depicts the incidence angle, both with respect to the substrate normal. *T*_H_ is the homologous temperature.

In addition to Al columns, further metallic columns are grown at 77 K substrate temperature and 82° incidence angle. Figure 4 gives an overview of the cross-sectional SEM images of those columns. A comparison of the images reveals that all deposited metals grow as high aspect ratio columns and therefore show overall similar morphologies. To understand why metals with different melting points show comparable morphologies if deposited at 77 K, the homologous temperature *T*_H_ is used as has also been done in previous research [16]. The homologous temperature *T*_H_ expresses the actual substrate temperature *T*_sub_ as a fraction of the melting point *T*_melt_ of the metal: *T*_H_ = *T*_sub_(K)/*T*_melt_(K). Due to the substrate temperature of 77 K, all deposited metals have a homologous temperature *T*_H_ between 0.02 (Ta) and 0.08 (Al). This corresponds to zone 1 in the structure zone model introduced by Movchan and Demchishin [19]. It is assumed that the shadowing length exceeds the surface diffusion length at low temperatures so that shadowing dominates the growth process, resulting in the formation of high aspect ratio columns. As a consequence, metallic tilted columns deposited at low temperatures (77 K) grow with similar morphologies since the growth process is dominated by shadowing.

Albeit all investigated metals grow with comparable morphologies at 77 K, a closer look at Figure 4 reveals differences in the diameter of the individual columns. For instance, columns of metals with high melting points such as Cr, Mo, and Ta (>2000 K) have smaller diameters (approximately 10 nm) than the columns of the remaining metals (e.g., up to 46 nm for Al). The detailed dependence of the adatom surface self-diffusion from the temperature is unknown for most metals, but it can be accepted that with increase of temperature the adatom surface mobility is also intensified since this is a thermally activated process which is exponentially related to the temperature. Consistently, a temperature rise from 77 K to 300 K leads for low-melting metals (e.g., Al) to a significant increase of the surface diffusion. In contrast, this temperature rise induces merely a small increase of the adatom diffusion on the surface for high-melting metals. It can be concluded that surface diffusion is expected to have a minor influence on the columnar morphology although the growth process is dominated by shadowing at 77 K substrate temperature.

### Column tilt angles

A closer comparison of the SEM images in Figure 4 reveals that the tilt angles β of the columns with respect to the substrate surface normal vary even though all columns are grown under the same experimental conditions (77 K substrate temperature and 82° incidence angle). For example, Ta columns have a much steeper tilt angle than Al and Ni columns, whereas the tilt angles for Cr, Mo and Ta columns are almost identical.

**Figure 5:**
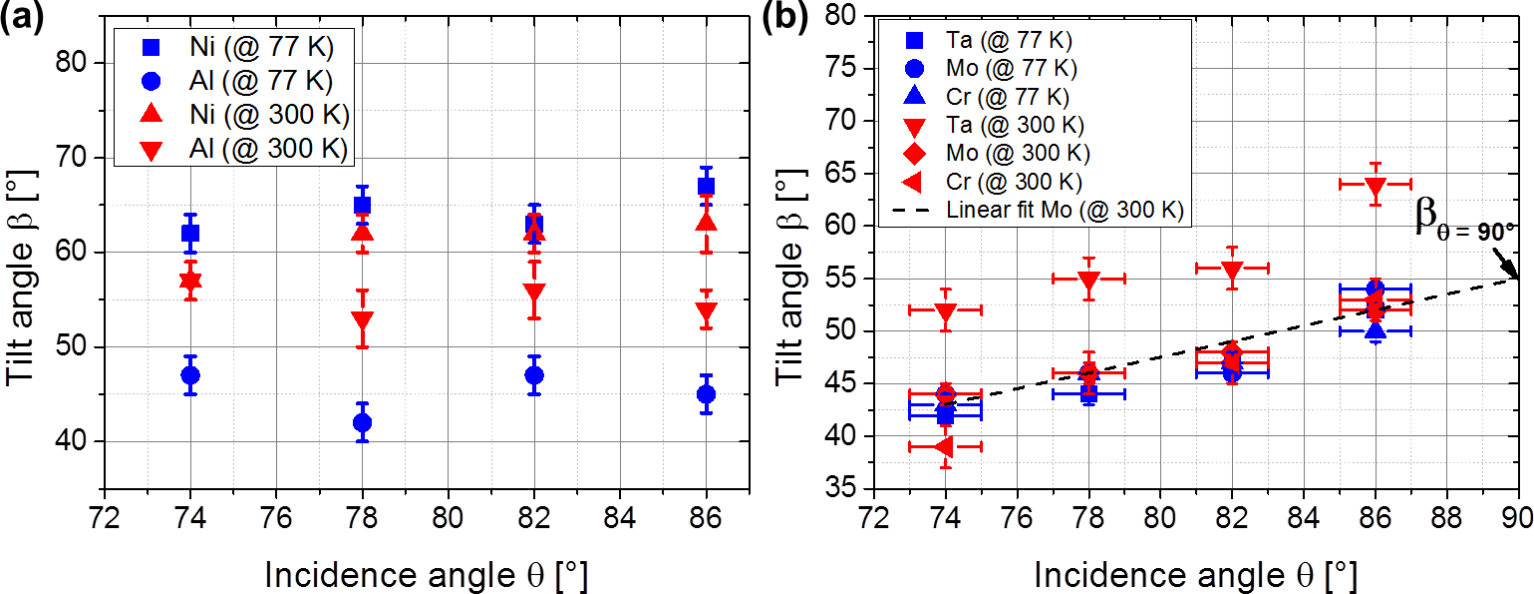
Column tilt angles of (a) low-melting metals Al, Ni and (b) high-melting metals Ta, Mo and Cr deposited at 77 K and 300 K substrate temperature and with varying incidence angles.

As shown in Figure 5a and 5b, respectively, the dependence of the tilt angle β on the incidence angle θ is studied exemplarily for Al and Ni as well as Cr, Mo and Ta deposited at 77 K (blue data points) and at 300 K (red data points) substrate temperature. Two general observations can be made. Firstly, for Ni, Cr and Mo columns the tilt angle for a fixed incidence angle does not change if deposited at 77 K or at 300 K. In contrast, the tilt of the Al and Ta columns becomes approximately 15° larger for deposition at 300 K. Secondly, the tilt angles of the Ta, Mo and Cr columns increases significantly with rising incidence angle, while the tilt angles of Al and Ni columns are not influenced remarkably by the incidence angle. It can be noted that between 77 K and 300 K, an influence of the incidence angle and of the substrate temperature on the columnar tilt angle is observed.

To understand the influence of temperature on the tilt angle β, this temperature relation is studied exemplarily for Ta, Mo and Al at homologous temperatures up to *T*_H_ = 0.3 for Ta (987 K substrate temperature), *T*_H_ = 0.3 for Mo (869 K substrate temperature) and *T*_H_ = 0.4 for Al (280 K substrate temperature). To balance statistical fluctuations of the measured data, the incidence angle θ is varied between 74° and 86° for each homologous temperature so that a relation between β and θ for each homologous temperature is found. These relations between β and θ are fitted linearly and are extrapolated to θ = 90°. Then, the corresponding tilt angle β at an incidence angle θ = 90° is determined (in the following called “β_θ=90°_”, see Figure 5b). This approach allows a parametrization of the relations between the angles β and θ for varying homologous temperatures and therefore facilitates to identify at which T_H_ these relations change remarkably.

**Figure 6:**
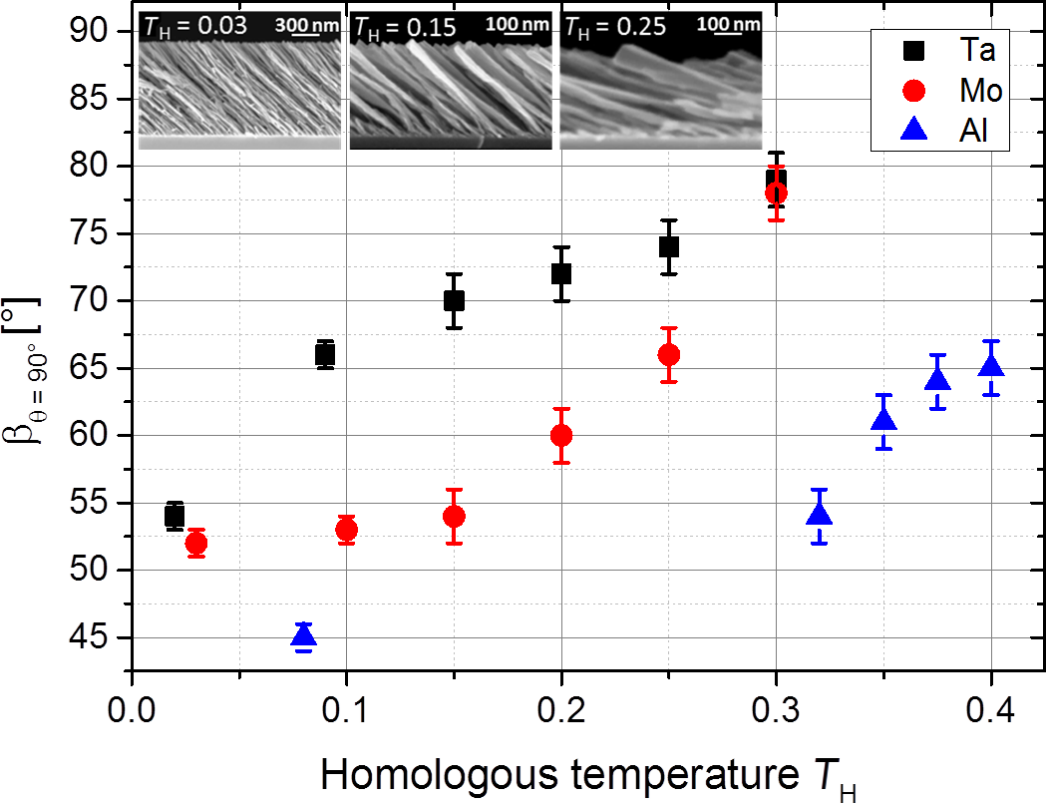
β_θ=90°_-values for Ta, Mo and Al columns depending on the homologous temperature (for explanation see text). Inset: Cross-sectional SEM images of tilted Mo columns grown at an incidence angle of 82° on thermally oxidized Si substrates at *T*_H_ = 0.03 (left), *T*_H_ = 0.15 (middle) and *T*_H_ = 0.25 (right).

Figure 6 illustrates the dependence of the β_θ=90°_ values on the homologous temperature for tilted Ta, Mo and Al columns. While the plot for the Mo columns shows a small slope up to *T*_H_ = 0.15, for larger *T*_H_ there is a significant increase up to *T*_H_ = 0.3. A reason is that below *T*_H_ = 0.15 the Mo adatoms have not enough energy to overcome the diffusion activation barrier. Thus, surface diffusion would be frozen until the homologous temperature exceeds *T*_H_ > 0.15 providing enough activation energy. As a consequence, Mo columns deposited at *T*_H_ = 0.03 and at *T*_H_ = 0.10 would have similar tilt angles, whereas for *T*_H_ > 0.15 a significant change in the tilt angles is expected. Indeed, the SEM images in Figure 6 (insets) show that the tilt angles become significantly larger for the Mo columns deposited at *T*_H_ = 0.25 compared to *T*_H_ = 0.03 and *T*_H_ = 0.10. Additionally, the calculation of the β_θ=90°_ values for Ta and Cr columns deposited at 77 K reveals that these are similar to the β_θ=90°_ values obtained for Mo columns. Since all three metals have large melting points (*T*_melt_ (Cr) = 2180 K, *T*_melt_ (Mo) = 2896 K and *T*_melt_ (Ta) = 3290 K), surface diffusion is assumed to be negligible at 77 K substrate temperature, which would account for the similar β_θ=90°_ values as shown in Figure 5b and Figure 6.

As mentioned above, the tilt of the Al and Ta columns is approximately 15° larger if deposited at 300 K (corresponding to *T*_H_ = 0.32 and *T*_H_ = 0.09, respectively). The plots for the Al and Ta columns in Figure 6 show that there is a significant increase of both slopes already for *T*_H_ > 0.08 and *T*_H_ > 0.02 (77 K), respectively. This is in contrast to the plot for Mo, where the slope rises significantly not until *T*_H_ > 0.15 (434 K). This means that for Al and Ta columns, surface diffusion is expected to be dominant already at 300 K, while for Mo columns a higher substrate temperature is required. Notice that previous research has shown that the metallic columns are crystalline. Since the adatom surface self-diffusion is expected to vary remarkably depending on different crystalline planes and on the local surface curvature, the description of the adatom movement on the columnar surface remains as a challenging task. However, there is only one measurement available concerning surface self-diffusion on Ta surfaces in the literature [20] so that it is not possible to discuss this issue adequately. Flahive and Graham [20] report about activation energies of 0.4 eV for Mo(110), 1.9 eV on Mo(111) and 1.4 eV on Mo(100), but Davydov [21] found a barrier of 1.2 eV on Mo(110), which is a three times higher value. The activation energies for self-diffusion on Al have been determined in more detail and scatter between 0.02 eV to 0.8 eV, depending on the surface and on the diffusion mechanism (hopping or exchange) [22–24]. A comparison between these reported data for Al and Mo shows that Al is expected to have smaller activation energies than Mo. This smaller activation energy corresponds to an enlarged surface self-diffusion so that the Al adatoms are also able to move in the shadowed regions. This reduces the shadowing effect as well as the influence of the incidence angle on the tilt angle (compare Figure 5a). Moreover, this smaller activation energy and in turn larger surface self-diffusion can also be a reason why the tilt angles are enlarged by approximately 15° if deposited at 300 K compared to 77 K.

To conclude, a consistent description of the relation between column tilt angle and the incidence angle for all investigated metals is not found. It is assumed that more detailed information concerning the surface self-diffusion on the metal surface planes would significantly contribute to the understanding of the column growth process. Further, the columnar tilt angle is expected to be the result of the complex interplay between surface self-diffusion, shadowing and changes in the crystalline structure of the deposited columns with varying substrate temperature, which should be considered (see previous section under Morphology and texture).

### Film porosity

The porosity of the thin metallic films is studied depending on the incidence angle and on the substrate temperature. Consider the case of vertical deposition (θ = 0°). Then, a compact layer with a film density of the deposited material ρ_θ=0°_ and a film thickness *t*_θ=0°_ (measured parallel to the substrate normal) is forming. This density ρ_θ=0°_ does not necessarily equal the bulk density of the material, since the film can contain inner voids, grain boundaries, etc. [25]. Tilting the substrate to an oblique angle θ leads to an oblique deposition geometry. Thereby, the effective area of the substrate is reduced by a factor of cos θ from the perspective of the incoming atoms. So, only a fraction of the incoming atoms will condense on the substrate, which results in a lower film thickness *t*_θ>0°_ compared to the vertically deposited film. Notice that the film thickness *t*_θ>0°_ is yet larger than expected, because shadowing is induced by the oblique deposition geometry so that the film density of the deposited material ρ_θ>0°_ is reduced significantly. According to [26], the porosity *P* of the nanostructured thin film can then be defined by the following equation:

[1]
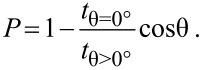


**Figure 7:**
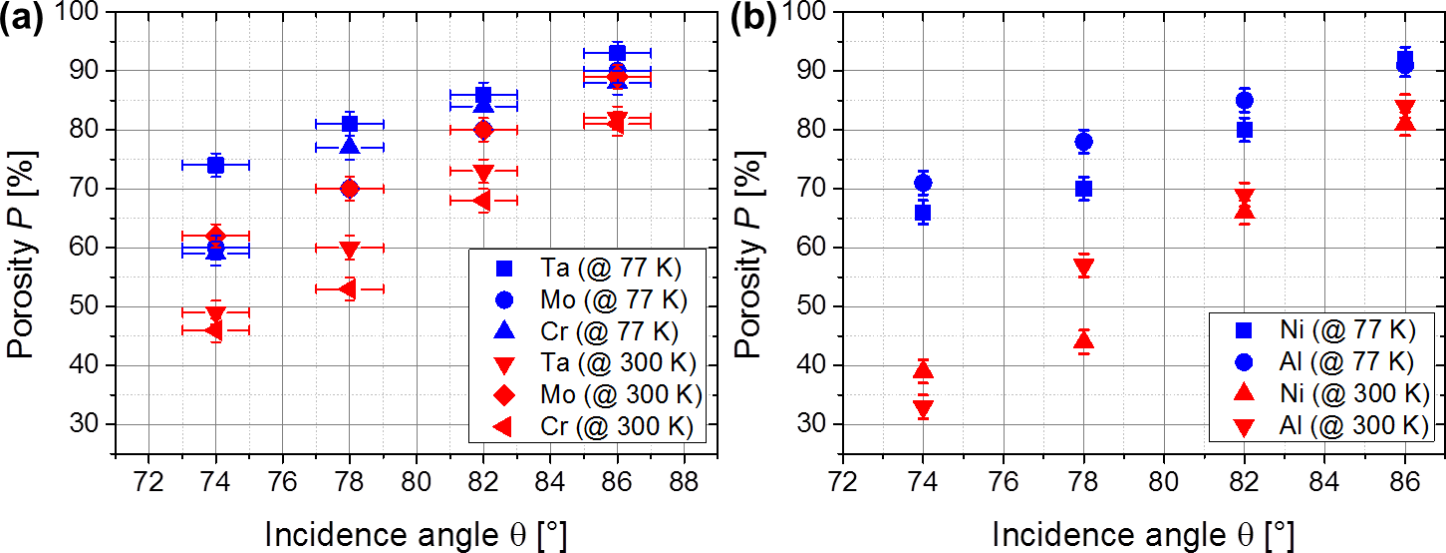
Porosity of (a) Ta, Mo and Cr and (b) Ni and Al thin films versus incidence angle.

Figure 7a and 7b depict the porosity of the obliquely deposited thin metallic films depending on the incidence angle for deposition at 77 K (blue data points) and 300 K (red data points). There are two general observations made. Firstly, it is observed that the porosity is enlarged for flatter incidence angles. In fact, increasing the incidence angle results in an enlarged shadowing length. Hence, more space is created between the tilted columns and this increases the porosity of the entire film. Secondly, all investigated metallic thin films show the tendency to grow more porous if deposited at 77 K compared to 300 K. This is expected because higher substrate temperatures are connected to increased surface diffusion, which enables the incoming atoms to condense in the shadowed regions and therefore favoring the growth of more compact thin films. For instance, the lowest porosities are observed for depositions at 300 K and at θ = 74° and reach values as low as 33% for Al. In contrast, thin Ta films deposited at 77 K and at θ = 86° show a porosity of 93%, which is an increase by a factor of approximately three. Such high film porosities have already been observed by Poxson et al. [26] for obliquely deposited SiO_2_. Further, Xi et al. [27] have also reported on a remarkably low refractive index for obliquely deposited SiO_2_, which corresponds to a film porosity of approximately 90%. In summary, both the incidence angle and the substrate temperature influence the porosity significantly. The highest film porosities for the investigated metals can be realized by depositing at low substrate temperatures in combination with highly oblique incidence angles.

## Conclusion

Al, Ni, Ti, Co, Cr, Mo and Ta tilted columns were successfully deposited under oblique deposition geometry at a substrate temperature of 77 K and at elevated temperatures. All tilted columns deposited at 77 K substrate temperature develop as high aspect ratio columns and therefore show a similar overall morphology. It is found that in this low temperature regime growth is dominated by shadowing, while surface diffusion has still a minor impact. A change in the crystalline texture is observed for Al columns deposited at 77 K compared to those deposited at 300 K. The thermally oxidized substrate does not determine the crystallites arrangement. Further, the relation between tilt angle and incidence angle depends strongly on surface diffusion, but a consistent description remains challenging due to the complex interaction between surface diffusion, crystallinity and shadowing. Moreover, it has been demonstrated that the porosity of obliquely deposited metallic thin films can be increased significantly by lowering the substrate temperature.

## Experimental

The metals Al, Ni, Ti, Co, Cr, Mo and Ta were evaporated by an electron beam. These metals were selected in order to cover a wide range of melting points (*T*_melt_ (Al) = 933 K to *T*_melt_ (Ta) = 3269 K). The pressure in the vacuum chamber was constant at approximately 10^−6^ Pa during deposition. In these OAD experiments the incidence angle θ was varied between 74° and 86°. Planar, thermally oxidized Si pieces with an 800 nm thick oxide layer were used as substrates. Substrate cooling down to 77 K was realized by a continuous flow of liquid nitrogen (LN2) through the Cu-block sample holder. The substrate temperature was controlled by two independent K-type thermocouples that were mounted directly on the surface of the LN2-sample holder. Another thermocouple of the same type was directly put into liquid nitrogen to ensure a precise calibration. During deposition with the LN2-sample holder, the substrate temperature was kept constant at 77 K with a fluctuation of ±1 K. In addition, another sample holder contains a heating system that enables the heating of the substrate up to a temperature of 1000 K. For all metals, the deposition rate was kept constant at 0.5 nm/s. The deposition rate and the deposited film thickness were controlled by a crystal quartz micro balance. The distance between crucible and LN2-sample holder was 35 cm, while this distance was 30 cm for the sample holder with the heating system. The samples were analyzed by scanning electron microscopy (SEM). Cross-sectional images of the samples were obtained by cleaving the samples before SEM-measurements. The tilt angles as well as the film height with respect to the substrate normal were determined manually. To provide a statistical reliability of the results, a considerably large number of measurements were performed. Moreover, the texture of the Al columns was analyzed by in-plane X-ray diffraction pole figure measurements using the in-plane degree of freedom of the detector arm and Cu Kα radiation [28]. The beam geometry in this commercial laboratory diffractometer was parallel.
